# Fungal community analysis by high-throughput sequencing of amplified markers – a user's guide

**DOI:** 10.1111/nph.12243

**Published:** 2013-03-28

**Authors:** Björn D Lindahl, R Henrik Nilsson, Leho Tedersoo, Kessy Abarenkov, Tor Carlsen, Rasmus Kjøller, Urmas Kõljalg, Taina Pennanen, Søren Rosendahl, Jan Stenlid, Håvard Kauserud

**Affiliations:** 1 Department of Forest Mycology and Plant Pathology, Swedish University of Agricultural SciencesBox 7026, SE-750 07, Uppsala, Sweden; 2 Department of Biological and Environmental Sciences, University of GothenburgBox 461, SE-405 30, Gothenburg, Sweden; 3 Institute of Ecology and Earth Sciences/Natural History Museum, University of Tartu46 Vanemuise St., 51014, Tartu, Estonia; 4 Department of Biology, University of OsloPO Box 1066, Blindern, N-0316, Oslo, Norway; 5 Department of Biology, University of CopenhagenØster Farimagsgade 2D, 1353, Copenhagen, Denmark; 6 The Finnish Forest Research InstitutePL 18, FI-01301, Vantaa, Finland

**Keywords:** 454-pyrosequencing, bioinformatics, barcoding, environmental sequencing, internal transcribed spacer (ITS) region, PCR

## Abstract

- Novel high-throughput sequencing methods outperform earlier approaches in terms of resolution and magnitude. They enable identification and relative quantification of community members and offer new insights into fungal community ecology. These methods are currently taking over as the primary tool to assess fungal communities of plant-associated endophytes, pathogens, and mycorrhizal symbionts, as well as free-living saprotrophs.
- Taking advantage of the collective experience of six research groups, we here review the different stages involved in fungal community analysis, from field sampling via laboratory procedures to bioinformatics and data interpretation. We discuss potential pitfalls, alternatives, and solutions.
- Highlighted topics are challenges involved in: obtaining representative DNA/RNA samples and replicates that encompass the targeted variation in community composition, selection of marker regions and primers, options for amplification and multiplexing, handling of sequencing errors, and taxonomic identification.
- Without awareness of methodological biases, limitations of markers, and bioinformatics challenges, large-scale sequencing projects risk yielding artificial results and misleading conclusions.

## Introduction

**Fig. 1:**
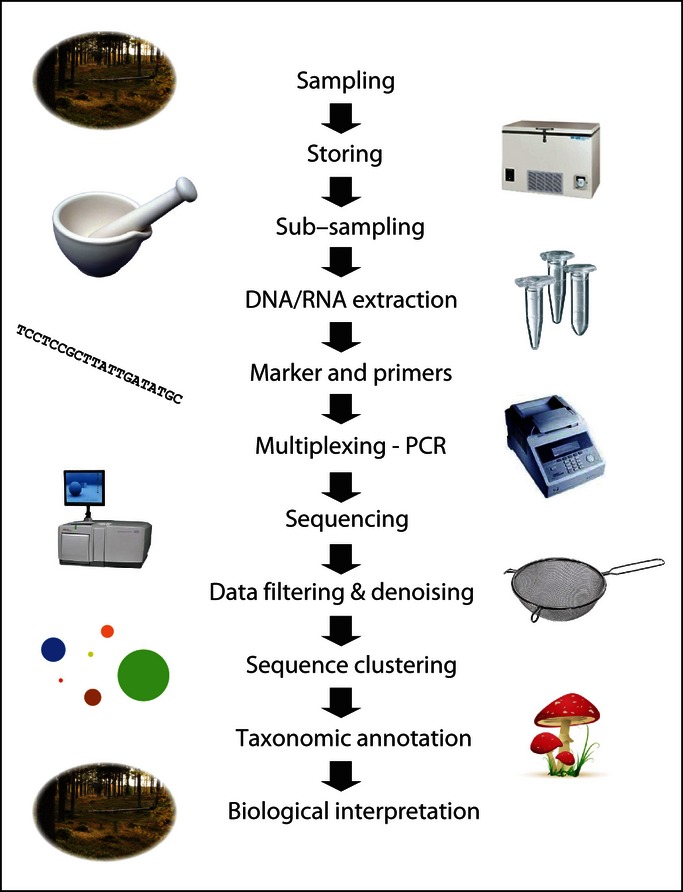
Overview of the steps involved in high-throughput sequencing of fungal communities.

The increasing use of molecular markers to identify fungi and analyse fungal communities in a phylogenetic context has initiated a boom in fungal ecology and phylogenetics. Our understanding of the important roles of fungi in symbiotic and pathogenic interactions with plants, as well as in transformation of plant litter and nutrient cycling, is thereby rapidly increasing. In particular, high-throughput sequencing methods enable detailed, semiquantitative analysis of fungal communities in large sample sets and provide ecological information that extends far beyond that provided by previous methods in terms of detail and magnitude. The process from field samples to species abundance data involves a long series of steps, from sampling via laboratory handling to bioinformatics treatment (Fig. 1). At each step, there is a risk of losing and distorting information. Here we present an overview of the steps involved, highlight potential pitfalls, discuss alternatives, and propose solutions.

## Sampling

The collecting of field samples to cover the targeted variation and enable statistically robust conclusions at the desired scale of inference represents a major challenge, and optimal strategies concerning the number and spatial distribution of samples have been discussed extensively (Petersen *et al*., 2005; Prosser, 2010; Lennon, 2011). Collection of fungal samples calls for some additional consideration, because of the indeterminate growth of mycelia and the multitude of contrasting morphologies and trophic strategies that coexist and interact in fungal communities.

Individual mycelia may sometimes reach metres or much more in size (Smith *et al*., 1992; Douhan *et al*., 2011), and to avoid spatial autocorrelation as a result of repeated sampling of single individuals, it is important to employ a minimum distance between samples that exceeds the largest expected size of fungal mycelia. For example, Lilleskov *et al*. (2004) found that, by keeping a minimum distance of 3 m between samples, most of the within-stand patchiness in ectomycorrhizal community composition (which presumably reflected the distribution of individual mycelia) could be avoided. It is also important to be aware that fungi are often antagonistic to each other, with mutual exclusion amplifying spatial variation at the scale of individuals (Boddy, 2000).

In forest soils with well-stratified profiles and deep organic layers, fungal communities may be more variable along vertical gradients than horizontally (Dickie *et al*., 2002; Lindahl *et al*., 2007; Baldrian *et al*., 2012). Free-living saprotrophs depend on recently dead (i.e. < 10 yr ago) organic materials with high energy content, and are therefore restricted to colonize recently deposited litter components close to the surface. By contrast, root-associated biotrophs may forage for nutrients in the more processed organic matter underneath, which has largely lost its value as an energy source (Lindahl *et al*., 2007). Thus, when soil cores span this vertical gradient, the integrated community composition may largely depend on the relative contribution of litter and rhizosphere material to the sample. Strong vertical stratification may be accounted for by subdivision of soil cores into well-defined horizons, preferably delimited by the structure and degree of decomposition of the material rather than by depth.

Fungal communities often display temporal variation in composition (Courty *et al*., 2008; Pickles *et al*., 2010; Davey *et al*., 2012), which may be short term in response to local weather events or cyclic in relation to seasons and the phenology of host plants. To analyse seasonal variations with statistical precision, repeated sampling should preferably stretch over time periods of several successive years.

## Handling of samples

Most markers in DNA-based community analysis are nuclear, and rapid multiplication of nuclei, for example in association with spore formation or rapid cell division of opportunists, may magnify the abundance of genetic markers without a corresponding major increase in biomass. Opportunistic growth is often induced by disturbances, implying that sampling may trigger rapid changes in DNA composition. For example, many soil fungi are intimately connected to plant roots, and disruption of root connections may induce death of root-associated species followed by rapid growth of mycelium-consuming opportunists (Lindahl *et al*., 2010). Sieving of soil samples leads to further release of readily usable substrates for opportunists. Thus, community development has to be arrested by freezing samples immediately upon collection, or at least slowed down by keeping samples cold until frozen at the earliest opportunity. Prolonged storage in the fridge should best be avoided, but freezing at −20°C should suffice to arrest community development and preserve DNA. Samples collected for RNA extraction have to be shock-frozen on dry ice or liquid nitrogen directly in the field, as RNA is prone to rapid degradation, and mRNA transcriptomes change in composition immediately upon disturbance. Samples intended for RNA extraction, as well as the extracted RNA, should be stored at −80°C, to ensure stable preservation. When direct freezing is not possible, chemical preservation may be an alternative (Grant *et al*., 2006). Preservation of samples by drying at room temperature is not a good option, because it involves incubation of moist samples at optimal temperatures for sporulation and rapid growth of opportunists. Freeze-drying enables long-term storage at room temperature, and may also aid later sample homogenization.

## Homogenization and subsampling

With some exceptions (see Taberlet *et al*., 2012), protocols for nucleic acid extraction are based on small amounts (mg to g) of sample material. Field samples are often much larger, and careful dispersion of tissues and aggregates is required to obtain small but still representative subsamples. The most commonly used techniques are bead beating and crushing in liquid nitrogen. Subsampling and homogenization have to be adapted to each specific substrate and study, but a basic rule is that, when the size of the subsample decreases in relation to the entire sample, careful homogenization becomes more critical. In a high-throughput sequencing study of ectomycorrhizal root systems, Kauserud *et al*. (2012) split samples after the homogenization step and observed a high consistency in fungal community composition of the independently analysed replicates, but large differences between repeated extractions have also been found (B. D. Lindahl *et al.,* unpublished). By including some technical replicates (i.e. split samples), the magnitude of stochastic effects and biases originating during subsampling, extraction, PCR, and sequencing may be assessed and put in relation to between-sample differences. If duplicate subsamples differ much in community composition, extraction protocols should be modified to allow larger sample sizes (e.g. in 50-ml tubes). When many samples are pooled, replication could even be implemented during sampling, so that two composite samples are collected and analysed from the same plot, providing information about stochastic variation associated with sampling. Should the sampling-associated noise threaten to overshadow more subtle treatment effects or ecological correlations, sampling effort would have to be increased.

After homogenization, new spatial structures may easily be created in the samples, for example by density fractionation at the slightest bumping. Ideally, subsampling should therefore be conducted by repeated subdivision rather than by a single ‘grab sampling’ (Petersen *et al*., 2005). When subsamples are small, community composition may be affected by stochastic sampling effects, as a result of the discrete nature of the sampled nuclei. This issue may be particularly problematic when screening for infectious propagules, such as resting spores, which may occur at low densities but still have a major ecological impact, for example as plant pathogens.

To ensure that fungal diversity is equally represented for all samples, DNA should be extracted from equivalent amounts of starting material. However, because densities may differ by orders of magnitude between different substrates, the distinction between volume- and weight-based quantification becomes important. For soil samples, determination of mass loss on ignition previous to extraction enables extraction from equal amounts of organic matter, which may be more relevant than total mass or volume.

## Extraction and purification

There are a multitude of methods and ‘ready-to-use’ kits available for extraction and purification of nucleic acids from field samples of different kinds, but they all rely on the same basic processes: (1) mechanical disruption of tissues, (2) solubilization of cell membranes by detergents under high salt concentrations, in order to release nucleic acids into solution and prevent electrostatic binding to contaminants, (3) removal of solid residues, (4) selective binding of nucleic acids to a solid matrix, or selective precipitation of nucleic acids and pelleting by centrifugation, (5) washing off of contaminants, and (6) elution/solubilization of nucleic acids.

Extraction protocols should yield high and uniform amounts of DNA, while the concentration of PCR inhibitors is minimized, so that optimal PCR conditions may be achieved. To avoid biases during sample preparation, the same DNA extraction protocol should ideally be used for all samples (Tedersoo *et al*., 2010a), although the relative efficiency of extraction methods may differ greatly between tissues and soil types (Martin-Laurent *et al*., 2001). For challenging substrates, such as forest soils with high humus content, a ‘raw extract’ produced by cell disruption and DNA precipitation may have to be further purified by binding of DNA to a silica matrix. Some of the problems with purity may be overcome by reducing the amount of starting material for DNA extraction. Counterintuitively, dilution of templates may often increase PCR yield as a result of release of inhibition (Wilson, 1997).

## Markers and primers

The ideal marker for fungal community studies should: have primer sites that are shared by all fungi, be of appropriate length for efficient amplification and sequencing, have high interspecific variation but low intraspecific variation, and be possible to align across all fungi. No known markers meet all these requirements. However, components of the nuclear ribosomal repeat unit (rDNA) are by far the most commonly used genetic markers for phylogenetic and taxonomic identification of microorganisms. The genes for the small subunit (SSU: 16S/18S) and large subunit (LSU: 23S/25S/28S) are juxtaposed and (in eukaryotes) separated by the internal transcribed spacer (ITS) region, which is transcribed but spliced away before assembly of the ribosomes. The ITS region is composed of two highly variable spacers, ITS1 and ITS2, and the intercalary 5.8S gene. This rDNA operon occurs in multiple copies in genomes, providing up to 100 times more DNA template from the same starting material than for single-copy genes (Herrera *et al*., 2009). The rDNA genes are highly conserved across large groups of organisms, making them ideal targets for general PCR primers that aim to amplify a wide range of taxa. However, amplified fragments must also contain enough variation to be informative at the phylogenetic level of interest. This is the main reason why the ITS region has been particularly attractive for mycologists. Because the ITS region does not code for ribosome components, it is highly variable; with a few exceptions (Gazis *et al*., 2011), even closely related species differ in sequence. At the same time, intraspecific variation is relatively low (Schoch *et al*., 2012). Intragenomic differences in ITS sequences have been detected in a few fungal taxa such as *Laetiporus* spp. (Lindner & Banik, 2011), but this does not seem to be a widespread phenomenon in Dikarya (D. L. Lindner *et al.,* unpublished). Using primers located in the adjoining ribosome-encoding genes or in the intercalary 5.8S gene, the ITS region may be amplified from a wide range of fungi. The choice of genetic marker also has to take the availability of reference databases into account, with ITS sequences having by far the best representation for Dikarya (Begerow *et al*., 2010). Thus, the ITS region was recently proposed as the formal barcode for fungi (Schoch *et al*., 2012). Although useful for species separation, the ITS region is too variable to address the phylogeny of higher ranks, that is, at the level of families and orders. When aiming to estimate phylogenetic distances across major fungal groups, the LSU provides an attractive alternative, being more conserved than the ITS and possible to align across distantly related taxa, yet also providing some resolution at lower taxonomic ranks (Porter & Golding, 2012). The more conserved SSU and LSU are widely used for Glomeromycota. In this phylum, single individuals may contain several divergent rDNA sequences (Sanders & Croll, 2010), and we have little knowledge of how the sequences obtained from field samples are distributed within mycelia and species.

In prokaryotes, the small subunit (16S) has been the prime target for phylogenetics and community analysis. Therefore, it may seem natural to use the corresponding SSU gene for fungi. However, in fungi and other eukaryotes, the SSU is more conserved than in prokaryotes (see Fig. 1 in Hartmann *et al*., 2010). Unless the focus is restricted to the highest phylogenetic ranks, that is, phyla and orders, the SSU gene provides little phylogenetic information and species delimitation power for Dikarya (Schoch *et al*., 2012), and is therefore not recommended as a target for species-level analysis of fungal communities. In many previous studies where the SSU was used as the target marker, conclusions were drawn at the level of species, based on perfect matches with database references but ignoring the fact that identical sequences could be found in hundreds of other species across entire orders of fungi. Identical SSU sequences may be shared between saprotrophs, parasites, and mycorrhizal fungi, because these ecological strategies have evolved repeatedly in relatively small phylogenetic lineages (Hibbett *et al*., 2000; James *et al*., 2006; Tedersoo *et al*., 2010b).

Protein-encoding genes usually occur as single copies in genomes, which may be advantageous for quantitative comparison of taxon abundances but disadvantageous during amplification. As a consequence of the nonconserved third base, protein-encoding genes contain more variation in the form of substitutions compared with deletions and insertions, enabling alignment across phylogenetically distant groups in spite of high variation in sequence. Furthermore, coding genes often contain introns with sufficient power for discrimination among species. A disadvantage with many protein-coding genes is that they occur in gene families where within-genome gene duplications often have taken place within the same time-frame as speciation, making the identification of gene orthologues problematic (Lindahl & Taylor, 2004; Bödeker *et al*., 2009). In addition, because of the nonconserved third base, it is difficult to design primers that cover all possible sequence variants, even when highly conserved functional domains are targeted.

Extraction of total DNA from environmental substrates may include material from dormant or even dead organisms, as free DNA may be preserved adsorbed to soil particles (Taberlet *et al*., 2012). By contrast, RNA has a shorter biological half-life ranging from minutes to hours (Kebaara *et al*., 2006). Transcribed messenger RNA (mRNA) which carries coding information of functional genes may be analysed to relate activity to specific gene products and eco-physiological functions (Kellner *et al*., 2010). However, because the functional and taxonomic annotation of genes is still far from completed and relatively few species are represented by their entire genomes in databases, the ribosomal genes remain the primary target of fungal community identification. Ribosomal RNA (rRNA) is quantitatively abundant and easily extracted from environmental samples (Pennanen *et al*., 2004), but the low phylogenetic resolution of these coding regions limits their use for species identification. Processing of transcribed rRNA to form mature ribosomes in eukaryotes includes splicing of the ITS regions, which is known to take place within a few minutes after transcription (Koš & Tollervey, 2010). This short window of time offers a possibility to amplify taxonomically valuable ITS sequences from newly transcribed RNA, reflecting very recent metabolic activity – even more recent than that indicated by SSU rRNA (reviewed by Rajala *et al*., 2011). The transient nature of ITS transcripts in the RNA pool makes them an attractive target when studying responses of fungal communities to short-term environmental fluctuations.

A multitude of primers have been designed and successfully applied to amplify fungal rDNA and rRNA from the environment. Most of these primers were originally designed to target fungi specifically, but turned out to amplify the DNA of other eukaryote lineages as well (e.g. ITS1–ITS5; White *et al*., 1990). The ITS1F primer (Gardes & Bruns, 1993) discriminates well against plants and has been widely used in analyses of plant-associated fungal communities. Primers such as ITS4B (Gardes & Bruns, 1993) and LB-W (Tedersoo *et al*., 2008) were designed with the aim of specifically targeting ectomycorrhizal fungi belonging to Basidiomycota. Hitherto, most primers have been constructed with amplification of monospecific samples (e.g. mycorrhizal root tips or pathogen-infected tissues) in mind. Nonbiased amplification of complex communities is more challenging, and competition for primers means that even single mismatches between primer and template impede or strongly bias amplification (Ihrmark *et al*., 2012). With the possible exception of the primers LR3/TW13 and LR5/TW14, which target highly conserved sites within the LSU, all fungus-specific and ‘universal’ primers inadvertently discriminate against specific fungal taxa (Bellemain *et al*., 2010). Thus, the choice of primer has a significant impact on how fungal communities are translated into amplicon communities. When the goal is to retrieve as many different fungi as possible, we recommend the use of primer combinations and primers with degenerate positions (i.e. mixtures of many different primers; e.g. Ihrmark *et al*., 2012; Toju *et al*., 2012). If primers with low specificity are used, nonfungal sequences may be removed at a later stage of the analysis. It should be noted, however, that when degenerate primers are used with high cycle numbers, depletion of specific primers in the mixtures may bias amplification in favour of species that match other, less depleted primers (Polz & Cavanaugh, 1998).

The length of the amplified fragments is a critical parameter that has to be considered when primers are chosen. Longer fragments contain more information for phylogenetic analyses. However, when aiming for minimized amplification biases, amplified fragments should be kept short, as increasing length of the target amplicon has a significant negative effect on assessments of microbial richness and biases community composition (Huber *et al*., 2009; Engelbrektson *et al*., 2010). With longer stretches of conserved sequence in the amplicons, the incidence of chimeric sequences also increases (Fonseca *et al*., 2012). By using primer sites in the 5.8S gene, amplification may be restricted to either the ITS1 or the ITS2 region only. Ihrmark *et al*. (2012) used new primers in the 5.8S gene to amplify 250–400-bp fragments containing the ITS2 region and found that diversity and community composition were much better preserved than when the entire ITS region was amplified. Additional primers with a similar purpose were designed by Toju *et al*. (2012). ITS1 and ITS2 share many properties, and similar results can be obtained with the two markers (Mello *et al*., 2011; Bazzicalupo *et al*., 2013). However, ITS2 is generally less variable in length compared with ITS1 and lacks the problem of co-amplification of a 5′ SSU intron that is common in many ascomycetes. The ITS2 has also relatively conserved secondary structure among eukaryotes, which potentially enables higher level phylogenetic comparisons and the use of ITS2 as a universal barcode across eukaryotic kingdoms (Coleman, 2009; Koetschan *et al*., 2010). Furthermore, ITS2 is somewhat better represented than ITS1 in databases (Nilsson *et al*., 2009).

With respect to Glomeromycota and other non-Dikarya lineages, it is more problematic to recommend primers, as we still lack information on the diversity in many groups. For Glomeromycota, a combination of taxonomically inclusive primers for nested PCR, involving the partial SSU, ITS, and partial LSU, has been elaborated (Krüger *et al*., 2009). Alternatively, a variable region of the SSU is amplified with the primers NS31-AM1, and the variable D2 region of the LSU is amplified with the primer FLR3 (aka glo454) in combination with either FLR4 or NDL22 (aka TW13) (van Tuinen *et al*., 1998; Gollotte *et al*., 2004; Lee *et al*., 2008; Öpik *et al*., 2009; Lekberg *et al*., 2012). Recently, other primers or primer combinations have been suggested (Lee *et al*., 2008; Stockinger *et al*., 2010), which also target regions in the SSU and LSU. The SSU has also been successfully used as a marker for Chytridiomycota (Freeman *et al*., 2009).

## Multiplexing

To make optimal use of high-throughput sequencing technologies, tagged amplicons from several samples may be mixed and sequenced in a single run. Sequences are then assigned to samples based on short sequence tags (i.e. molecular identifiers – MIDs), which are unique to each sample. In addition, most high-throughput sequencing methods require that amplicons are fitted with specific adaptor sequences. The adaptor sequences, as well as the tags, can be incorporated into the PCR primers, but they may also be added by ligation to the PCR products. Three options are available (Table 1).

1. Both adaptors and sample tags are included in the PCR primers (Jumpponen & Jones, 2009). This method enables directional sequencing, which is beneficial if the fragments are too long to be sequenced throughout their entire length. However, in some labs such long primer constructs (> 45 bp) has proved to impair PCR efficiency and to cause problems with primer dimerization (Wallander *et al*., 2010). Such problems may be ameliorated by a nested PCR approach, where ordinary primers are used during most of the PCR and the extended primers are added during the last few cycles (Kauserud *et al*., 2012), but such complicated PCR schemes may increase the risk of contamination and distortion of relative abundances.
2. Sample tags are included in the PCR primer but adaptors are added to the PCR product by ligation (Ihrmark *et al*., 2012). This method reduces the length of primers to < 30 bp, and PCR may be conducted using standard programmes. With adaptors added by ligation, amplicons will be sequenced in random orientation. This may cause problems for long amplicons, where sequences from different ends may have no or only partial overlap. Nondirectional sequencing also implies that half of the sequences have to be reversed before further analysis. Furthermore, the 5′-end nucleotides of the tags may interfere with ligation, so that certain samples are favoured in the final mix (Ihrmark *et al*., 2012). This problem may be overcome by fitting all sample tags with the same 5′-end nucleotide or by adding more PCR product from certain samples.
3. Both adaptors and sample tags are ligated onto PCR products. Here, the same standard primers may be used for all samples, but PCR products from different samples have to be kept separated through ligation. When many samples are analysed, this method increases work-load and costs considerably.

**Table 1:** Different options for the addition of sample tags and sequencing adapters to PCR products

1	Primers	ADAPTOR - TAG - PRIMER		PRIMER - TAG - ADAPTOR
	PCR	ADAPTOR - TAG - PRIMER	-----------------	PRIMER - TAG - ADAPTOR
2	Primers	TAG - PRIMER		PRIMER - TAG
	PRC	TAG - PRIMER	-----------------	PRIMER - TAG
	Ligation	ADAPTOR - TAG - PRIMER	-----------------	PRIMER - TAG - ADAPTOR
3	Primers	PRIMER		PRIMER
	PCR	PRIMER	-----------------	PRIMER
	Ligation	ADAPTOR - TAG - PRIMER	-----------------	PRIMER - TAG - ADAPTOR

Berry *et al*. (2011) found indications that tag-extended primers may introduce biases in community composition and advised that tagged primers are added during the last PCR cycles. However, in later tests of different tags on artificially assembled communities (Ihrmark *et al*., 2012), tag-related biases were marginal. It is, however, important that the two nucleotides at the 3′ end of the tag do not match with corresponding nucleotides in the target priming site, which would allow the tag to act as an extension of the primer, potentially causing positive amplification bias. Switching of sample tags after pooling of separately amplified PCR products may have an impact on sequencing results and lead to numerous false positives as a result of cross-contamination (Carlsen *et al*., 2012). To be able control for this phenomenon, amplicons may be tagged at both ends. In order to minimize the risk of misidentification of sequence tags, it is also important that all tags differ from each other by at least two nucleotides (Parameswaran *et al*., 2007; Faircloth & Glenn, 2012).

## PCR

Preservation of genotype composition through DNA extraction and subsequent PCR amplification is a major challenge. The number of PCR cycles has to be minimized, as excessive cycles may result in preferential amplification of rare sequences as well as the creation and further propagation of chimeric sequences (Kanagawa, 2003; Haas *et al*., 2011). Particularly when degenerate primers are used, the PCR should preferably be interrupted while in the exponential phase (Polz & Cavanaugh, 1998). The cycle number may be reduced by optimizing extraction protocols and by choosing markers and primers that yield short amplicons and, thereby, increase PCR efficiency. Generally, one should aim for weak to medium-strong PCR products, as visualized on an electrophoresis gel.

Different polymerases tend to differ in fidelity, and choosing a high-fidelity polymerase will reduce the number of nucleotide incorporation errors produced during PCR amplification. For instance, Phusion® (New England BioLabs Inc., Ipswich, MA, USA) and *Pfu* Ultra™ (Agilent Technologies Inc., Santa-Clara, CA, USA) both have a 50× higher fidelity than *taq* (Li *et al*., 2006). With a *taq* error rate of 2.3 × 10^−5^, the proportion of amplicons with error for a 250-bp fragment amplified through 30 cycles of PCR will be 0.3% for Phusion® and *Pfu* Ultra™ and 16% for *taq*. However, the majority of these errors would be caused by a single bp difference only and could be accounted for during denoising and sequence clustering. Choosing a high-fidelity enzyme may also reduce the number of recombinant (chimeric) amplicons (Lahr & Katz, 2009).

Quantitative real time PCR (qPCR) is a valuable tool when optimizing extraction protocols and PCR conditions. In a qPCR cycler, the increasing product concentration may be followed for each individual reaction during the entire cycling programme. Thus, qPCR may be used to pre-screen samples, adjusting cycle numbers to ensure that the PCR is interrupted during the phase of exponential increase in product concentration. Extraction yield, template dilution, and PCR parameters can be optimized, and PCR inhibition may be assayed by spiking samples with standard template. Real-time PCR may also be used to quantify the amount of template, that is, the absolute number of extractable copies of marker genes per amount of extracted substrate (Baldrian *et al*., 2013). By choosing primers with different specificities, the total amount of fungal DNA or individual taxa may be quantified. However, several technical replicates are required, in order to gain precision in the estimates. It is also critical to control for PCR inhibition and template availability, preferably by spiking samples with standard reference DNA before extraction.

The need to employ the most stringent discipline during preparation of samples for community sequencing cannot be emphasized enough. Negative controls (blank extractions) should always be included in all PCR reactions (Tanner *et al*., 1998). However, when the number of PCR cycles is increased, PCR products will inevitably form, also in negative controls, unless all laboratory work is conducted under rigorously sterile conditions; a single spore that falls into a PCR tube is enough to yield a band on the gel. This is another reason to aim for high template concentrations and low cycle numbers, so that the effects of minute contaminations on overall community composition are minimized. There is also the possibility to include positive controls in the form of a simple, standard ‘mock community’ of known qualitative and quantitative composition (c.f. Ihrmark *et al*., 2012).

## Purification, quantification, and pooling of PCR products

Before sequencing, PCR products from different samples are mixed in equimolar proportion, so that the DNA sequence output is evenly distributed across all samples. It may also be beneficial to pool several PCR reactions from each sample, in order to even out stochastic distortion of community composition during PCR (Polz & Cavanaugh, 1998; Ihrmark *et al*., 2012). Before pooling, PCR products have to be purified, to remove primers and short DNA fragments. If long composite primers are used, this step may require particular attention, especially when primer dimerization is a problem. In difficult cases, gel excision may be a solution, but this approach involves excessive laboratory work when sample numbers are large. When establishing the concentration of PCR products, methods based on fluorescent DNA-binding dyes have higher resolution than methods based on UV absorbance, particularly as many types of sample tubes may release UV-absorbing compounds from the plastic (Lewis *et al*., 2010). If PCR products are available in excess, specially designed normalization plates are available, which retain the same amount of DNA from each sample and discard the surplus. To ensure a high quality of the sample, that is, absence of primers and fragments of unwanted sizes, and firmly establish the final amounts of DNA, the combined size fractionation and concentration measurements offered by the Bioanalyzer technology (Agilent Technologies Inc.) are useful. When running the protocols for the first time, confirmatory Sanger sequencing of a few cloned amplicons is recommended before high-throughput sequencing, particularly if complex PCR schemes are employed.

## Sequencing platforms

In 2005 the first high-throughput sequencing platform from 454 Life Sciences (Branford, CT, USA) was introduced to the market (Margulies *et al*., 2005), and *c*. 3 yr later the first fungal ecology studies were published based on this technology (Buee *et al*., 2009; Jumpponen & Jones, 2009; Öpik *et al*., 2009). The 454-sequencing technique is routinely used both for shotgun sequencing of genomic DNA/cDNA and in-depth sequencing of PCR amplicons. A typical run on the GS FLX+, using titanium chemistry, takes 1 d and yields 1–1.5 million reads with a length of *c*. 400–500 bases, which are ideal read lengths for covering either ITS1 or ITS2 (plus primers and tags). Longer read lengths, up to 1200 bases, have recently been generated. Ion Torrent (i.e. the Ion PGM Sequencer; Life Technologies, Carlsbad, CA, USA), which was introduced to the market in 2011, has similarities with the 454 technology but measures released protons (pH) directly rather than light. The major advantages of Ion Torrent are its short run-time (*c*. 2 h), high yields, and a competitive price compared with 454-sequencing. According to the manufacturer, the new Ion Proton Sequencer may generate 60–80 million 200-base reads (with the Proton I chip). The shorter read lengths have hitherto made Ion Torrent unsuitable for the analysis of the ITS region, but up to 400-bp sequences have recently been generated. Illumina sequencing (Illumina Inc., San Diego, CA, USA) is currently the most successful and most widely adopted next-generation sequencing platform, but has hitherto not been adopted for analysis of fungal communities because of limited read lengths. However, according to the manufacturer, paired-end sequences on the MiSeq platform now enable 2 × 250-base read lengths and a yield of *c*. 30 million reads. The sequencing platform SOLiD (Life Technologies) also results in a high number (up to 4.8 billion) of short reads. As a consequence of their reduced costs and tremendous yields, both Ion Torrent and Illumina MiSeq will obviously challenge the Roche 454 technology. So-called ‘third-generation sequencing platforms’ are based on single-molecule and real-time sequencing, with the first platform, PacBio RS, introduced in 2011 by Pacific Biosciences (Menlo Park, CA, USA). Read lengths may be up to several kb but the sequence quality and output are still too low for diversity analyses based on amplified markers. However, by sequencing the template several times (circular consensus sequencing), reads of high quality may be produced. Other upcoming techniques that will probably have a substantial impact on the field are based on registering the DNA (or RNA) as it goes through nanopores placed in artificial membranes. For comprehensive reviews of current and future sequencing technologies, see Glenn (2011) and Shokralla *et al*. (2012). Further up-to-date news on this rapidly developing topic may be found at http://seqanswers.com.

## Bioinformatics analysis

As PCR errors become visible when sequences are based on single molecules of PCR product, and high-throughput methods also generate frequent errors during the sequencing procedure, data sets derived by high-throughput sequencing must be subjected to extensive quality control measures (Kunin *et al*., 2010). The same data set analysed using only read-score-based filtering versus more advanced filtering methods may differ around five-fold in the number of derived operative taxonomic units (OTUs; Quince *et al*., 2009). ITS sequences seem to be particularly prone to 454-sequencing errors, presumably because of the high incidence of homopolymers, that is, repetitions of a single nucleotide, which are a major source of error in 454-sequencing (Balzer *et al*., 2011).

The read-score-based base-pair pruning applied by the sequencing factory is at best a poor replacement for sequence quality management programs such as ampliconnoise (Quince *et al*., 2011), the Denoiser implemented in qiime (Reeder & Knight, 2010), dada (Rosen *et al*., 2012) and acacia (Bragg *et al*., 2012), which are all tailored for high-throughput sequencing data. ampliconnoise also supports detection of sequence chimeras, whose presence otherwise would inflate diversity estimates significantly (Fonseca *et al*., 2012). 454-sequencing data sets may contain a nontrivial number of sequences that represent primer dimers, seemingly random sequence data, or gene segments other than the one targeted (Balzer *et al*., 2011). It may happen, as in the study by Wallander *et al*. (2010), that as much as 95% of 454-sequencing reads have to be excluded because of quality-related issues, but 20–40% is a more common figure. Whether or not a sequence represents the ITS region can be established using ITSx (http://microbiology.se/software/itsx/), which uses hidden Markov models and the hmmer package (Eddy, 2011) to detect the flanking SSU, 5.8S and LSU genes. The ITS1 and ITS2 as well as the full ITS region can then be extracted automatically from the sequence data set depending on which genes were detected. A similar tool for the SSU was released by Bengtsson *et al*. (2011). Diversity estimates also depend on the amount of sequences derived from samples, and one way to reduce bias associated with different numbers of reads in the different samples is to randomly subsample all samples down to the size of the smallest sample (Gihring *et al*., 2012).

Establishment of sequence similarities requires alignment of sequences. When global alignments are possible, as is the case for less variable markers, such as the LSU and SSU, data may be entered into commonly used pipelines developed for general microbial ecology, such as mothur (Schloss *et al*., 2009). Global alignment also enables analysis of the phylogenetic distance between communities, using tools such as UniFrac (Hamady *et al*., 2010), in which the difference between communities depends not only on which members are included, but also on how closely related they are. However, for the ITS region, methods based on global alignments are impractical, because of high variability in sequence and length, and clustering of ITS sequences usually has to depend on pairwise alignments. Pairwise alignments require major computational capacities, and several available bioinformatics pipelines specially developed for processing of fungal ITS data sets derived by 454-sequencing, including clotu (Kumar *et al*., 2011), scata (http://scata.mykopat.slu.se), and plutof (Abarenkov *et al*., 2010a), run as web-based tools on high-capacity computer clusters.

**Fig. 2:**
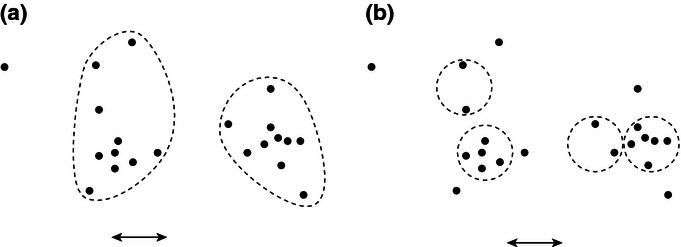
Illustration of (a) single-linkage clustering and (b) complete-linkage clustering of the same objects in a two-dimensional space. Arrows indicate the clustering threshold distance. With the same clustering threshold distance, single-linkage clustering yields fewer clusters and fewer singletons.

During clustering, sequences sharing a predefined level of similarity are assembled into OTUs. Complete-linkage clustering (furthest neighbour) yields OTUs that can be thought of as circular; with a 97% similarity threshold, all sequences within a cluster will be at most 3% different from each other. With single-linkage clustering (nearest neighbour), a 97% similarity threshold means that it is enough that a sequence is at most 3% different from any other sequence in the OTU to be included in that OTU, and OTUs tend to be amoeboid rather than circular (Fig. 2). blastclust (http://ftp://ftp.ncbi.nih.gov/blast/) is an example of a single-linkage clustering program, and clotu, scata, plutof, and mothur all feature single-linkage clustering. Complete-linkage algorithms (e.g. uclust; Edgar, 2010) are sensitive to the choice of seed sequences, which typically relies on sequence frequencies or length. This is not a concern for single-linkage clustering, which is deterministic, that is, the same OTUs are arrived at irrespective of seed sequence. The ‘greedy clustering’ of single-linkage methods, where clusters expand until there are no similar sequences left to enter, makes them efficient in the handling of sequencing errors (Huse *et al*., 2010), and single-linkage clustering may to some extent replace more computationally intensive quality management programs. However, single-linkage clustering requires that OTUs are phylogenetically well separated from their neighbours, or there is a major risk that they merge into large clusters (a ‘snowballing effect’). Furthermore, it is important to consider that local alignments over subsections of sequences usually lead to higher pairwise similarities than global alignments, and local alignments with a low match-length threshold may yield overly large clusters.

An alternative to pairwise comparisons, which may be attractive when computational capacity is limited, is to compare sample sequences to a set of identified reference sequences, for example, with mafft (Katoh & Frith, 2012) or qiime (Caporaso *et al*., 2010; https://github.com/qiime/its-reference-otus). This method is best suited for inventories of well-described taxa, but has obvious limitations when exploring less charted parts of the fungal kingdom.

An increasing number of clustering programs apply other similarity measures than absolute distances, relying on, for example, the grammatical structure of the sequence data (notably gramcluster by Russell *et al*. (2010), crop by Hao *et al*. (2011), and crunchclust by Hartmann *et al*. (2012)).

crunchclust, clotu, and scata are notable in offering a pyrosequencing homopolymer collapse option, where OTUs cannot be delimited based on differences in homopolymer regions alone. Furthermore, both clotu and scata allow easy checking for noncompatible tag combinations when tags are fitted at both ends of amplicons. Sequence clustering produces the best results when sequences of comparable coverage are employed, and the user should consider running tools, such as ITSx (http://microbiology.se/software/itsx/) or v-xtractor (Hartmann *et al*., 2010; Kerekes *et al*., 2013) for SSU and LSU, to ensure that the query sequences are at least roughly comparable in terms of coverage of the target region.

To assign taxonomic affiliations to the sequences obtained is a major challenge, and projects should be planned both with ample time for data analysis in mind and in such a way that bioinformatics and taxonomic expertise is accounted for among the project members. Following the sequence clustering step, the derived OTUs are typically examined for taxonomic affiliation through BLAST-based similarity searches in the INSD or UNITE (Abarenkov *et al*., 2010b) databases or, for LSU sequences, through a Bayesian classifier (Liu *et al*., 2012). We recommend the use of the most common sequence in each OTU as a basis for taxonomic examination (less favoured options include the longest sequence in each OTU or the consensus sequence). The INSD sequence corpus is in part compromised by the presence of incorrectly annotated, chimeric, or otherwise substandard entries, and the user is well advised to use the UNITE (ITS; Abarenkov *et al*., 2010b; Tedersoo *et al*., 2011), SILVA (SSU; Pruesse *et al*., 2007) or MaarjAM (SSU; Glomeromycota; Öpik *et al*., 2010) databases instead. UNITE maintains a downloadable copy of the fungal ITS sequences in INSD (http://unite.ut.ee/repository.php), and this copy is subject to third-party annotation and other quality management measures. More than 75 000 INSD sequences have been annotated, including the exclusion of *c*. 1000 chimeric entries and the taxonomic annotation and re-annotation of 13 500 entries, and for local similarity searches, this data set is much to be preferred over a raw dump of the INSD.

Complications associated with the taxonomic affiliation of sequences based on BLAST searches are discussed in Christen (2008) and Kang *et al*. (2010). Although dependent on settings, BLAST searches tend to favour long sequence, and the presence of conserved sequence segments in the query sequence, and anyone analysing high-throughput derived ITS sequences may want to prune any large parts of the SSU, LSU, and possibly also the 5.8S from their ITS sequences before doing similarity searches. It should be recognized that proper sequence-based identification involves delimitation of taxa and requires understanding of fundamental phylogenetics as well as a basic taxonomic overview of the fungal kingdom (Nilsson *et al*., 2008). There are countless examples of how blind reliance on best BLAST hits for identification may lead in totally wrong directions. The construction of a ‘rough’ phylogenetic tree, based on a crude alignment and neighbour joining of sample and reference sequences, may aid understanding of the material in a phylo-taxonomic context. For a schematic overview of the phylogenetic composition and diversity across samples, BLAST results can be imported and viewed in the program megan (Huson *et al*., 2011). Based on the consistency of the top BLAST matches, the sequences will be mapped at different levels in a predefined taxonomy (e.g. the GenBank taxonomy). The scata pipeline approaches OTU identification in a different way, with database references and sample sequences clustered together. The reference sequences included in each OTU are listed in the data output, allowing assignment of taxonomic identities.

Another alternative to BLAST for taxonomic assignment is provided by the naïve Bayesian classifier method (Liu *et al*., 2012) implemented in the ribosomal database project (Wang *et al*., 2007). Starting from a large training set of well-annotated reference sequences, the Bayesian classifier attempts to assign query sequence to the various taxonomic levels offered by the reference sequences. It computes a bootstrap value for each assignment, thus providing a rough measure of confidence of the assignment at each level. Its accuracy is comparable to, or somewhat better than, that of BLAST, and it is substantially faster than the latter. A potential downside of the LSU classifier is the limited number (and taxonomic scope) of the public fungal LSU sequences. However, given the more conserved nature of the LSU compared with the ITS region, LSU sequences from previously unsequenced lineages are typically still assignable to higher taxonomic ranks such as order or class, which is not always the case with ITS sequences.

Finally, it is important that data are stored in a publicly accessible way, and that the bioinformatics handling of data is properly accounted for in publications (Nilsson *et al*., 2011). An extensive list of bioinformatics resources can be found in Bik *et al*. (2012).

## Data interpretation

In spite of denoising and the use of ‘greedy’ clustering algorithms, high-throughput data sets usually contain a large number of singletons (unique sequences present only once in the data set) that deviate to varying degrees from the original template. As such erroneous singletons inflate diversity, a common practice has been to remove them before downstream statistical analyses (Tedersoo *et al*., 2010a), but of course many singletons may represent authentic, rare taxa (Kauserud *et al*., 2012). The abundance of artificial singletons in high-throughput data sets makes estimates of total sample diversity by endpoint extrapolation of rarefaction curves risky. As the incidence of erroneous singletons increases with sequencing effort, species accumulation curves tend to increase infinitely (Quince *et al*., 2009). This implies that the relevance of diversity estimators, such as Jackknife and Chao indices, which rely on the abundance of singletons and doubletons relative to more common OTUs, may be questioned for high-throughput sequencing data (Dickie, 2010). It remains uncertain to what extent this problem can be ameliorated by proper bioinformatics procedures.

The reliability of OTUs with a low number of sequences may also be questioned, and a conservative approach has been to remove all clusters with less than, for example, five reads. However, the appropriate cut-off level for removing ‘low-frequency clusters’ depends on the total number of sequences per sample and the clustering parameter settings. If the primary aim of studies is to investigate community–environment relationships or effects of experimental treatments rather than estimating alpha diversity or screening for rare taxa, it has been found that pruning of rare OTUs has a marginal effect on subsequent multivariate statistical analyses (Gobet *et al*., 2010). By contrast, particular attention has to be paid to the validity of rare OTUs when data are analysed based on presence/absence. As false positives may occur as a result of tag switching (Carlsen *et al*., 2012), and even the slightest cross-contamination may have a major impact, we recommend pruning of OTUs with low numbers of sequences. Such pruning should preferably be carried out on a per-sample basis, as an OTU that is common in one sample may occur as a low-abundant contaminant in other samples.

To what degree high-throughput sequencing data can be used quantitatively is much debated (Amend *et al*., 2010; Baldrian *et al*., 2013). When interpreting community analyses based on molecular markers, it is important to remember that abundance of genetic markers in extracts does not reflect biomass in the samples. Amplification of an artificial community assembled from PCR products showed that community structure may be fairly well conserved through PCR and 454-sequencing, provided that the amplicons are short and primers match with all species in the community (Ihrmark *et al*., 2012). By contrast, the quantitative composition of an artificially assembled spore community was not well reflected by 454-sequencing in the study of Amend *et al*. (2010), suggesting that diverging numbers of rDNA repeats in different species in combination with differences in extractability may lead to severe quantitative biases. Furthermore, accurate quantification of genomes in a sample does not suffice to describe taxonomic biomass distribution; species with long, filamentous cells are likely to be underrepresented, whereas fungi with yeast-like growth and/or small cells may be overrepresented, because of their high nucleus to biomass ratio.

## Concluding remarks

New high-throughput methods outperform earlier approaches in terms of resolution and magnitude and offer unprecedented insights into fungal community ecology. However, without awareness of methodological biases, limitations of markers or bioinformatics challenges, large-scale sequencing risks yielding artificial results and misleading conclusions. Thus, early claims of astonishingly high species richness in 454-sequenced amplicons were exaggerated, because of problems in distinguishing technical artefacts from true diversity. Although more sophisticated bioinformatics tools are now available, high-throughput assessment of species richness remains a major technical challenge. Furthermore, considering that even a species represented by a single spore would be recorded in a sufficiently deeply sequenced sample, the biological relevance of such assessments may be questioned. Absolute analyses of species presence and diversity are also sensitive to contaminations during sampling, laboratory processing and sequencing. We argue that the major benefit of high-throughput methods rather lies in the capacity to provide information about the main fungal colonizers in large numbers of samples, to a progressively decreasing cost in terms of money and laboratory labour. In the near future, automated processing of samples may increase the scope and statistical power of ecological studies even further. In addition, novel sequencing techniques continually increase data output, which in combination with rapidly expanding databases of entire genomes enables a development away from molecular markers and PCR amplification towards direct analysis of meta-genomes and meta-transcriptomes of complex fungal communities (Kuske & Lindahl, 2013).
